# The impact of Climate Change on the Western Pacific Subtropical High and the related ozone pollution in Shanghai, China

**DOI:** 10.1038/s41598-019-53103-7

**Published:** 2019-11-18

**Authors:** Luyu Chang, Jianming Xu, Xuexi Tie, Wei Gao

**Affiliations:** 1 Department of Atmospheric and Oceanic Sciences, Fudan University, Shanghai, 200438 China; 2 Shanghai Typhoon Institute, Shanghai Meteorological Service, Shanghai, 200030 China; 3 Shanghai Key Laboratory of Meteorology and Health, Shanghai, 200030 China; 4 Key Laboratory of Aerosol Science and Technology, SKLLQG, Institute of Earth Environment, Chinese Academy of Sciences, Xi’an, China; 5 Center for Excellence in Regional Atmospheric Environment, Chinese Academy of Sciences, Xiamen, 361021 China; 6 Anhui Province Key Laboratory of Atmospheric Science and Satellite Remote Sensing, Hefei, 230000 China

**Keywords:** Atmospheric chemistry, Environmental impact

## Abstract

Severe ozone (O_3_) episodes occur frequently in Shanghai during late-summers. We define geopotential height averaged over the key area region (122.5°E-135°E, 27.5°N -35°N) at 500 hPa as a WPSH_SHO_3_ index which has high positive correlation with surface O_3_ concentration in Shanghai. In addition, the index has a significant long-term increasing trend during the recent 60 years. Analysis shows the meteorological conditions under the strong WPSH_SHO_3_ climate background (compared to the weak background) have several important anomalies: (1) A strong WPSH center occurs over the key area region. (2) The cloud cover is less, resulting in high solar radiation and low humidity, enhancing the photochemical reactions of O_3_. (3) The near-surface southwesterly winds are more frequent, enhancing the transport of upwind pollutants and O_3_ precursors from polluted regions to Shanghai and producing higher O_3_ chemical productions. This study suggests that the global climate change could lead to a stronger WPSH in the key region, enhancing ozone pollution in Shanghai. A global chemical/transport model (MOZART-4) is applied to show that the O_3_ concentrations can be 30 ppbv higher under a strong WPSH_SHO_3_ condition than a weak condition, indicating the important effect of the global climate change on local air pollution in Shanghai.

## Introduction

With rapid industrialization and urbanization in recent decades, China has been experienced persistent and serious air pollution problem, causing important impacts on human health and ecological environment (like crop damages)^1–5^. Shanghai, as a rapidly developing megacity in China with a population of over 20 million, is suffering severe ozone pollutions during summer and haze episodes during winter in recent years^6–8^. The monitoring data in Shanghai demonstrates that the ozone concentrations had a significant long-term increasing trend during recent years^9,10^, and the monthly mean O_3_ concentrations increased about 67% from 2006 to 2016. In 2017, ozone pollution duration (148 days yr^−1^) exceeded PM_2.5_ pollution duration (60 days yr^−1^), becoming a primary pollutant for affecting air quality in Shanghai.

In the troposphere, ozone is produced by a complicated chemical process, which initializes by the photochemical reactions of ozone precursors, such as nitrogen oxides (NO_X_) and volatile organic carbons (VOC_S_)^11–13^. As the increase in industrial activity and number of automobiles, the precursors of ozone (O_3_) and the global budget of oxidization are also significantly increased^14,15^. Although photochemical process is important to determine ozone concentrations, previous studies suggest that local meteorological parameters such as temperature, solar radiation, relative humidity, horizontal wind speed and direction, and cloud cover also play important roles in controlling ozone concentrations. For example, elevated ozone concentration is usually accompanied with strong solar radiation and small winds, which is favorable for the photochemical production of ozone and the accumulation of ozone and its precursor^16^. Tie *et al*. (2009) suggest that radiation, wind speed and wind direction are the most important meteorological factors for causing the variability of surface ozone concentrations in Shanghai. These factors directly affect the photochemical reaction, regional transportation and diffusion process of ozone. Furthermore, synoptic-scale weather pattern, with spatial scale less than 1000 km, is also an important factor in controlling ozone variability^17–21^. For example, anticyclones (i.e., high pressure systems) produce favorable conditions for ozone production. At the center of anticyclones, it is normally sunny weather, with low wind velocity, causing high O_3_ production and accumulation^22,23^. In coastal cities, sea-land breeze can be an important meteorological factor to affect ozone distributions. Tie *et al*. (2009) finds that the impact of sea breeze on O_3_ concentration is noticeable in the city of Shanghai under calm weather condition.

It is well known that the West Pacific Subtropical High (WPSH) is evident as a semi-permanent, sub-tropical anticyclone high pressure over the western North Pacific, affecting the summertime weather and climate in China^24–26^. Despite of the effect on weather conditions (such as summertime precipitation and temperature), WPSH has also important effects on vegetation coverage and other research field^27,28^. Currently, the impact of the position and intensity of the WPSH on summertime ozone pollution over eastern China have been paid more attention. Most of the previous studies examine the relationship between the WPSH and ozone on a daily scale. For example, He *et al*.^29^ focus on an short-term ozone pollution event in Shanghai and finds that ozone mixing ratios in summertime at Chongming (a surface site in the northeast of Shanghai) are often higher during the days when the center of the WPSH locates to the southeast of that site, with a weak intensity. Some results^30^ show the subsidence air caused by the WPSH plays a crucial role in the formation of high-level O_3_. Zhao *et al*.^31^ studies the impact of WPSH on surface ozone daily variability over eastern China and demonstrates that a stronger WPSH is associated with lower ozone in South China but with higher ozone in North China, suggesting that this south-north difference can be explained by changing moisture transport associated with the WPSH variability.

Generally, the WPSH is closely associated with the timing and spatial distribution of summer ozone concentrations in East Asia and may intensify in a warming climate background^32^. However, there is s lack of study to analyze the effect of global changes on WPSH and the consequence on the O_3_ concentrations in the Shanghai region. The focus of this study is to investigate a strong inter-annual variability of ozone pollution and the effect of global climate change on summertime ozone pollution in Shanghai. The paper is organized as follows: in Section 2, we describe the information of the methodology. In Section 3, some results and analysis are analyzed. Section 4 shows a brief conclusion of the results.

## Discussion

**Figure 1:**
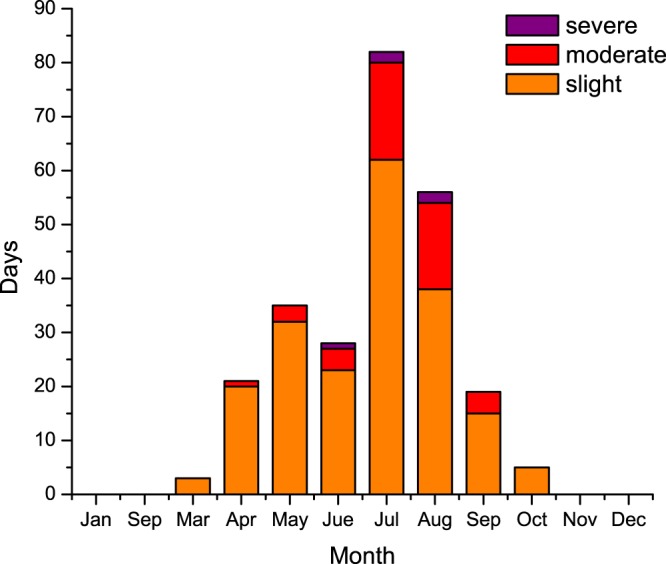
Monthly variation of ozone pollution days in Shanghai during 2013 to 2017 (orange, red and purple columns represent slight, moderate and severe pollutions respectively). The O_3_ concentrations were measured by the Chinese National Environmental Monitoring Center (CNEMC) in the Shanghai area. The maps were generated by origin software version 8.0.

Gao *et al*. (2017) and Lin *et al*. (2017) have demonstrated that the ozone concentrations in Shanghai steadily increase, with a strong seasonal variation. Because the effect of WPSH on O_3_ concentrations often occurs in summer time, the monthly variation of ozone during 2013 to 2017 in the Shanghai region is analyzed and is presented in Fig. 1. Figure 1 shows that the 8h-averaged ozone (MDA8 O_3_) concentrations have highest occurrences in late-summer (July and August) compared with those in other months. The new Chinese national ambient air quality standards (CNAAQS2012, GB 3095–2012) has defined the severe, moderate, and slight ozone pollution^33^. The total pollution days on July and August from 2013 to 2017 are 82 and 56 days, respectively. According to the study by Gao *et al*. (2017), the ozone concentrations are low in early summer (June), because of the occurrence of frequent precipitation in June which is named Meiyu-Baiu-Changma rain belt in China. Previous studies^34^ have demonstrate that the seasonal northward shifts of the WPSH are closely associated with the onset and withdrawal of the EASM, during July and August, the ridge of WPSH locate around Yangtze-Huaihe River region, and the weather conditions here are strongly controlled by the main body of WPSH system. As a result, it is important to understand the impact of WPSH on surface ozone in Shanghai during late-summer.

**Figure 2:**
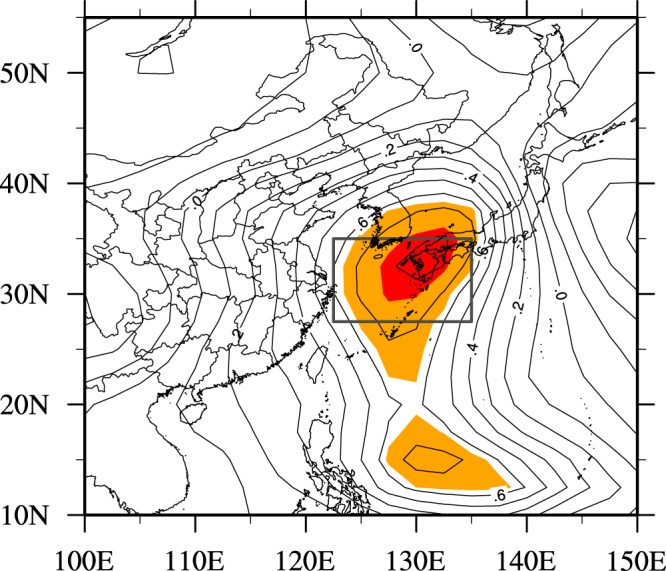
The distribution of correlation coefficients between monthly mean MAD8 O_3_ in Shanghai and the 500 hPa geo-potential height over Eastern Asia during late-summer from 2013 to 2017 (Black box represents the key region of WPSH. Values of red and orange shades have passed positive 95% and 99% confidence levels based on student-t test, respectively). NCEP Reanalysis data provided by the NOAA/OAR/ESRL PSD, Boulder, Colorado, USA, from their Web site at https://www.esrl.noaa.gov/psd/. The map was generated by NCL software [The NCAR Command Language (Version 6.3.0) [Software]. (2015). Boulder, Colorado: UCAR/NCAR/CISL/TDD. 10.5065/D6WD3XH5].

As we all know, WPSH is a multi-dimensional climate system, its temporal and spatial circulation characteristics are more complex, so it is often simplified by one dimension monitoring index to analyze its effect on ozone. Five operational indices of WPSH based on the 5880 geopotential height (gpm) at 500 hPa, including the area, intensity, ridge lines, northern extension, and western boundaries are announced monthly by the National Climate Center (NCC) in China to describe the WPSH’s evolution^35^. But the correlations between ozone concentrations in Shanghai and indices on a monthly scale is very poor, Consequently, it is necessary to develop a new objective index, which is not only with clear physical meanings to objectively describe the location and intensity of the WPSH, but also has significant associations with surface ozone in Shanghai. Figure 2 shows the distribution of correlation coefficients between monthly mean MAD8 O_3_ in Shanghai and the 500 hPa geopotential height over Eastern Asia during late-summer from 2013 to 2017. To obtain correlation analysis data as more as possible, monthly mean data are used rather than late-summer mean data. Correlations between ozone concentrations and 500 hPa geopotential height are positive in most parts of Eastern Asia. The significant positive correlation coefficients with 500 hPa geopotential height (exceeding the 95% confidence level based on student-t test) are located over eastern ocean of China. The significant correlation coefficients related to the geopotential height averaged over the key area region (122.5°E-135°E, 27.5°N-35°N) at 500 hPa shows a maximum positive correlation coefficient (0.78). Thus, we define geopotential height averaged over the key area region at 500 hPa as the new definition index of WPSH’s effect on ozone (called WPSH_SHO_3_). It not only represents the activity center and intensity of the WPSH over the key area region, but also it has high positive correlation with the surface ozone concentration in Shanghai.

**Figure 3:**
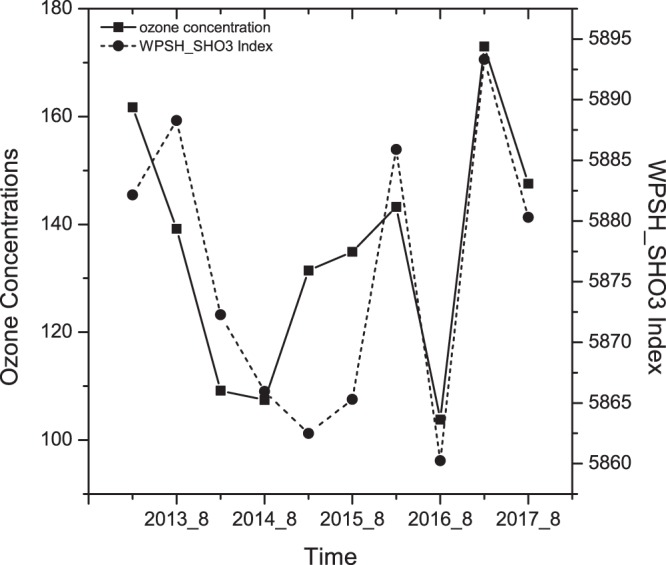
The inter-annual variability of monthly mean MAD8 O3 and WPSH_SHO3 index in Shanghai during late-summer from 2013 to 2017. The O_3_ concentrations were measured by the Chinese National Environmental Monitoring Center (CNEMC) in the Shanghai area. The map was generated by origin software version 8.0.

Figure 3 displays the inter-annual variability of monthly mean O_3_ (MAD8) in Shanghai and the corresponding WPSH_SHO_3_ index from 2013 to 2017. The characteristics of the two parameters were highly correlated (except during 2015), with a correlation coefficient of 0.78 (exceeding the 99% confidence level). The high correlation between WPSH_SHO_3_ index and O_3_ concentrations indicated that the intensity of the WPSH_SHO_3_ can be used as an indictor to predict a general tendency of late-summer O_3_ concentrations in Shanghai.

**Figure 4:**
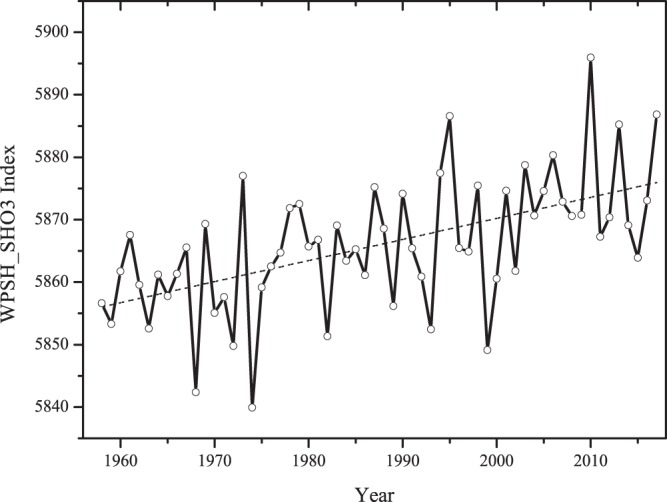
The long-term trend of midsummer mean WPSH_SHO3 index from 1958 to 2017. NCEP Reanalysis data provided by the NOAA/OAR/ESRL PSD, Boulder, Colorado, USA, from their Web site at https://www.esrl.noaa.gov/psd/. The maps were generated by origin software version 8.0.

West Pacific Subtropical High (WPSH) is mainly affected by large-scale circulations, previous studies^36,37^ have shown different results of how the ongoing global warming would change the WPSH. To understand the impact of Climate Change on the WPSH and its impact on ozone pollution in Shanghai, Fig. 4 shows the long-term trend of late-summer mean WPSH_SHO_3_ index from 1958 to 2017. As shown in Fig. 4, the index of WPSH_SHO_3_ significantly increased during the recent years (1958–2017), with an increasing rate of about 5 gpm decade^−1^. For example, the WPSH_SHO_3_ was about 5885 gpm in 2017 and 5855 gpm in 1958, respectively. This rapid increase suggested that the global climate change had strong impacts on the WPSH_SHO_3_, which might have important effects on air pollutants (such O_3_) in Shanghai (as shown in Fig. 3).

To systematically study the impact of WPSH_SHO_3_ on O_3_ concentrations in Shanghai during late-summers, two different late-summer cases were selected and compared. The first case (Jul. 2017) was a typical strong WPSH case, with the maximum anomalies geopotential height, while the second case (Aug. 2016) was a typical weak WPSH, with the minimum anomalies geopotential height. It is interesting to note that the difference of geopotential height between the two cases is about 30 gpm, which is equivalent to the value between the values of WPSH_SHO_3_ in 1957 and 2017 (see Fig. 4). As a result, comparing the two extreme cases can provide useful insights to understand the impact of global climate change on WPSH_SHO_3_ and the corresponding effects on O_3_ concentrations in Shanghai.

**Figure 5:**
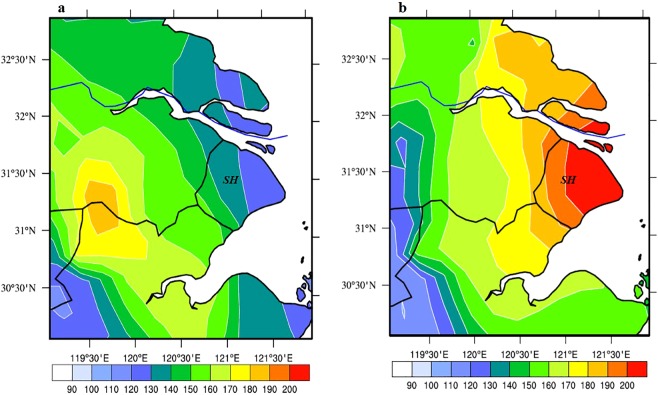
Monthly mean O_3_ (MAD1) concentration in Shanghai and its surrounding areas in the weak WPSH case (Aug. 2016) (**a**), and the strong WPSH case (Jul. 2017) (**b**). (The ozone data which is measured by the Urban Air Quality Center in China has been interpolated to a rectilinear grid using bilinear interpolation). The O_3_ concentrations were measured by the Chinese National Environmental Monitoring Center (CNEMC) at 367 monitoring stations and were interpolated to grid data by using iterative improvement objective analysis. The maps were generated using NCL software [The NCAR Command Language (Version 6.3.0) [Software]. (2015). Boulder, Colorado: UCAR/NCAR/CISL/TDD. 10.5065/D6WD3XH5].

Figure 5 shows the monthly mean O_3_ (MAD1) concentrations during the strong (Jul. 2017) and the weak (Aug. 2016) WPSH_SHO_3_ cases in Shanghai and its surrounding regions. The results show that there was significantly difference for the O_3_ concentrations in these 2 cases. During the weak case, the maximum O_3_ concentration was located in the inland and the west of Shanghai (SH), with a highest value of >180 μg m^−3^. As a result, the O_3_ concentrations in SH were low, ranging from 120–140 μg·m^−3^. In the contrast, during the strong WPSH case, the maximum O_3_ concentration was higher than the weak WPSH case, and the highest O_3_ located in the city of Shanghai (SH), with a highest value of >200 μg m^−3^. As a result, the O_3_ concentrations in SH were high, ranging from 180–200 μg m^−3^, resulting in a large anomaly of O_3_ concentrations (60 μg m^−3^) between the strong and the weak WPSH cases in Shanghai.

To understand the impact of WPSH_SHO_3_ on the anomalies of ozone concentrations between strong and weak WPSH cases, detailed meteorological conditions were analyzed.

**Figure 6:**
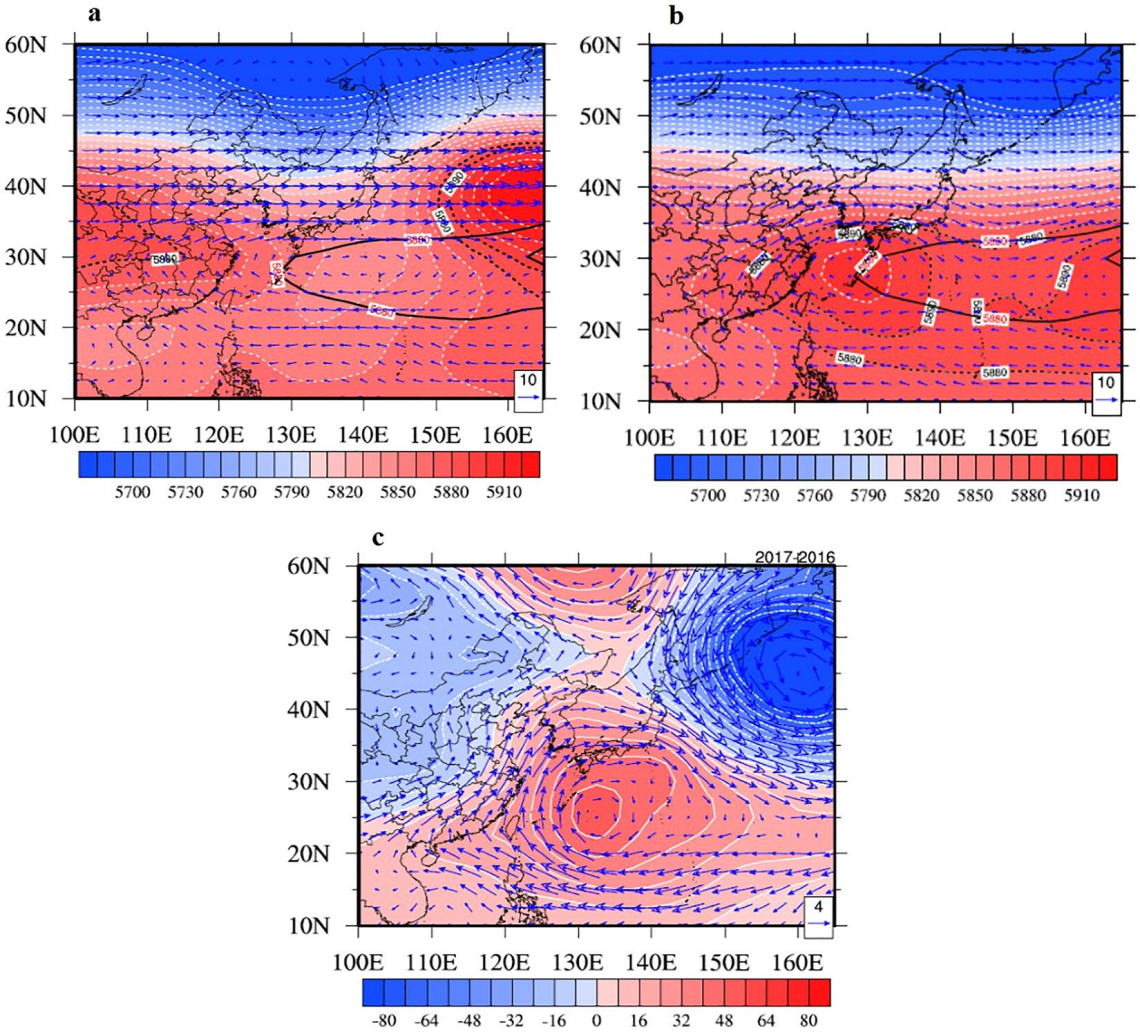
The actual mid-level (500 hPa) circulations in Aug. 2016 (**a**) and Jul. 2017 (**b**) and its anomalies (**c**) (dash lines represent actual WPSH_SHO_3_, solid line represents climatological (1988–2017 mean) WPSH_SHO_3_, the arrows represent the wind vector and the actual geopotential height are shaded). NCEP Reanalysis data provided by the NOAA/OAR/ESRL PSD, Boulder, Colorado, USA, from their Web site at https://www.esrl.noaa.gov/psd/. The maps were generated by NCL software [The NCAR Command Language (Version 6.3.0) [Software]. (2015). Boulder, Colorado: UCAR/NCAR/CISL/TDD. 10.5065/D6WD3XH5].

Figure 6 shows the large-scale atmospheric circulations over 500 hPa between the two cases. It shows the mid-level (500 hPa) circulations of weak case (Fig. 6a) and strong case (Fig. 6b) and its anomalies (the monthly mean values in 2017 minus the monthly mean values in 2016, shown in Fig. 6c). Compared to climatological WPSH_SHO_3_ (solid lines), the WPSH_SHO_3_ had characteristics with bigger area and stronger intensity under strong WPSH_SHO_3_ climate background (Fig. 6b). Moreover, there was significant sub-center of WPSH_SHO_3_ over the key area regions (Fig. 3). In contrast, the weak WPSH_SHO_3_ had no high center over the West Pacific Ocean (Fig. 6a). As a result, there were significant positive anomalies of geopotential height over 500 hPa in the West Pacific Ocean region, which was approaching the key area region mentioned in Fig. 2. For example, the geopotential height over 500 hPa in the key area region of strong case is about 30–50 gpm higher than weak case.

Tie *et al*. (2009) find that radiation and wind (speed and direction) are the most important meteorological factors for affecting surface ozone pollution in Shanghai, which directly affect the photochemical reaction, diffusion and transport of surface ozone. In this study, the anomalies of the large-scale circulations over 925 hPa, relative humidity, solar radiation, and surface daily-max temperature between the weak and strong cases are investigated.

**Figure 7:**
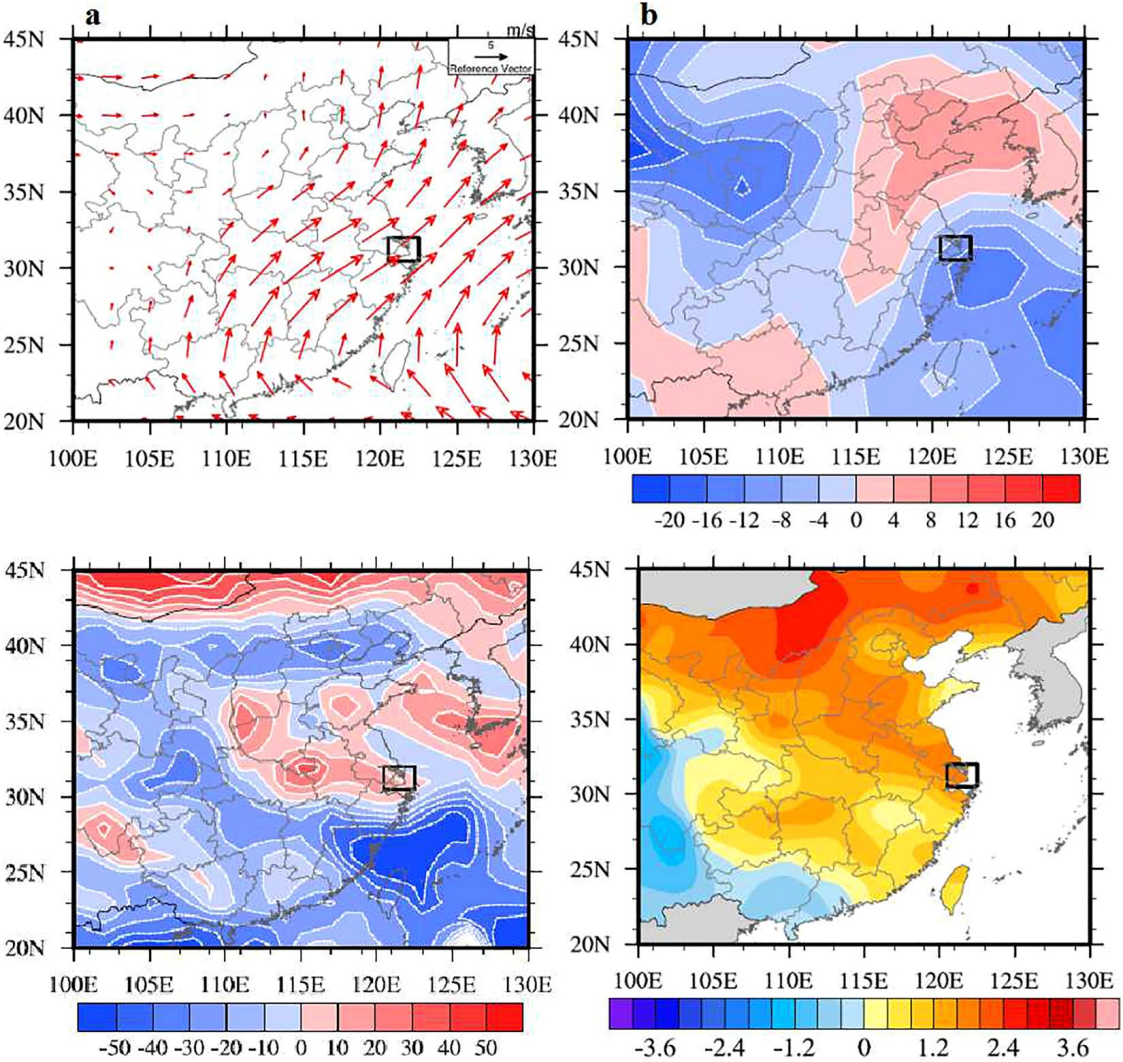
Anomalies of monthly mean low level circulation (**a**), low level relative humidity (units: %) (**b**), solar radiation (units: W·m-2) (**c**), and surface daily-max temperature (units: °C) (**d**) between Jul. 2017 and Aug. 2016 (high minus low). Black box covers the area of Shanghai. NCEP Reanalysis data provided by the NOAA/OAR/ESRL PSD, Boulder, Colorado, USA, from their Web site at https://www.esrl.noaa.gov/psd/. The maps were generated by NCL software [The NCAR Command Language (Version 6.3.0) [Software]. (2015). Boulder, Colorado: UCAR/NCAR/CISL/TDD. 10.5065/D6WD3XH5].

As shown in Fig. 7a, in the strong case (July 2017), there was is a strong anomalous southwesterly winds in Shanghai, which was enhanced by 4–5 m·s^−1^ over 925 hPa. The surface temperature was higher 3 °C than the weak case (Fig. 7d). The relative humidity was weaker by 20% than the weak case (Fig. 7b), and the radiation was stronger by 20 W•m^−2^ than the weak case in Shanghai (Fig. 7d). During the southwesterly winds condition, the upwind region is a large-scale forest. As a result, the enhanced southeasterly winds transported upwind biogenic emissions to Shanghai, causing and the increasing the ozone pollution in Shanghai^38^. However, it’s essential to give some supplementary explanation that although the anomaly of specific humidity was small between the strong case and the weak case, the local solar radiation and temperature were enhanced during the strong case (Fig. 7c,d). As a result, the higher solar radiation and temperature reduced relative humidity (RH). Furthermore, the meteorological conditions, with high temperature, low humidity, and high solar radiation were favorable for producing high-level O_3_ episodes^39^. And the possible effects of relative humidity (RH) on the ozone formation can be well explained by Yu (2018), as the paper shows, more air humidity could inhibits O_3_ formation by lowering air temperature and some complicated chemistry processes, like decreasing the chain length of peroxy radical chemical amplifiers (HO_2_, RO_2_, and RC(O)O_2_), and decreasing the chain length of NO_2_ by enhancing particle water, and destroys the existing O_3_ photo-chemically by water vapor through catalytic O_3_ destruction cycle^40^. As a result, during the strong WPSH_SHO_3_ case, the photochemical production of O_3_ was more active than the weak case.

**Figure 8:**
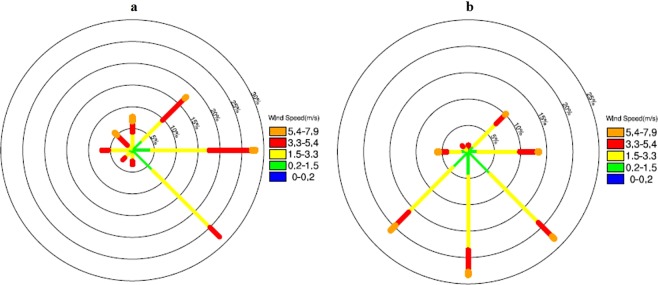
The wind rose maps in the weak case (**a**) and the strong case (**b**) over Baoshan station (color bars represent different wind speed). The wind direction and wind speed data were measured at Shanghai Baoshan Climate Observatory. The maps were generated using NCL software [The NCAR Command Language (Version 6.3.0) [Software]. (2015). Boulder, Colorado: UCAR/NCAR/CISL/TDD. 10.5065/D6WD3XH5].

Same characteristics are found in local weather conditions in the Baoshan station in Shanghai. Table 1 and Fig. 8 show the local weather conditions in the city of Shanghai. The average meteorological conditions were calculated for the both strong and weak cases in the Baoshan observation station. As shown in Table 1, the relative humidity was lower in the strong case (65% compared with the 71% in the weak case). The hourly maximum temperature was higher in strong case (30 °C) than the weak case (32 °C). The measured photolysis rate of NO_2_ (J[NO_2_]) was higher (1.9 × 10^–3^ s^−1^) than the weak case (1.1 × 10^−3^ s^−1^), producing higher ozone photochemical production. Another important factor that increases the O_3_ concentrations was due to the wind directions. In the strong WPSH_SHO_3_ case, southwest wind occurred frequently in Shanghai. In the contrast, in the weak WPSH_SHO_3_ case, east and southeast winds were dominated (Fig. 8), which transported relative clean air from ocean, resulting in lower O_3_ concentrations in the weak case. In addition, the areas with subtropical high-pressure control (the strong WPSH_SHO_3_ case) are dominated by subsidence air flow, producing weak convection and cumulus clouds. Under less cloud conditions, solar radiation is high, which may greatly enhance the photochemical reactions of O_3_.

**Table 1:** Local weather conditions of the strong WPSH_SHO_3_ and weak WPSH_SHO_3_ conditions in the Baoshan station.

	RH (%)	Tmax (°C)	J[NO_2_] (10^−3^ s^−1^)	Wind speed (m s^−1^)
2016	71.3	29.8	1.1	2.7
2017	65.4	32.4	1.9	2.5

The J[NO_2_] was measured at the Pudong monitoring site of Shanghai Meteorological Service and other data were measured at Shanghai Baoshan Climate Observatory.

In order to better investigate the effects of the WPSH_SHO_3_ on the O_3_ pollution in Shanghai, a global chemical transport model (MOZART-4) is applied to calculate the distribution of ozone concentrations under the different WPSH_SHO_3_ conditions (the strong case in Jul. 2017 and the weak case in Aug. 2016). The detailed model description is shown in section 2.3. First we conduct a model validation by comparing the model result with the measurement.

**Figure 9:**
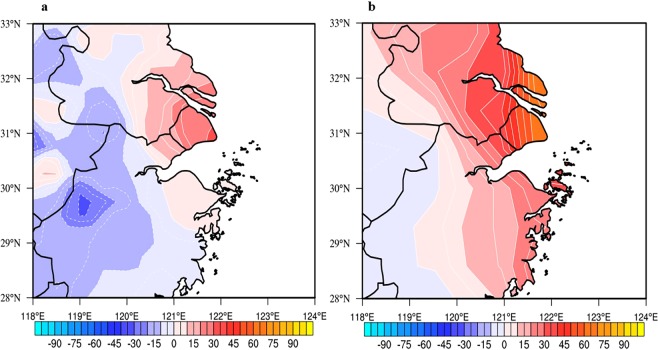
The O_3_ concentration (μg·m^−3^) anomalies in the Shanghai region in the strong and weak cases (Jul. 2017 and Aug. 2016), which were derived from observation data (**a**) and MOZART-4 (**b**). The maps were generated using NCL software [The NCAR Command Language (Version 6.3.0) [Software]. (2015). Boulder, Colorado: UCAR/NCAR/CISL/TDD. 10.5065/D6WD3XH5].

Figure 9 shows the comparisons between the model results of MOZART-4 and the observed anomalies of monthly mean O_3_ concentrations between Jul. 2017 and Aug. 2016. The results suggest that the observations and the simulated results present a similar in the spatial distribution. For example, there were positive O_3_ anomalies in Shanghai and the northwest of Shanghai, while there were negative O_3_ anomalies in the west of Shanghai. However, the magnitude anomalies were higher in the simulation than the measured results. In Shanghai, both measured and modeled O_3_ showed strong positive anomalies, ranging from 20 to 60 μg·m^−3^. Because this study is focus on the O_3_ anomalies in Shanghai, the model simulation provides a base for the further studies.

Because there are several important factors in controlling the O_3_ concentrations, such as diffusion (DIF), (b) advection (ADV), and (c) gas-phase chemistry (CHEM), model analysis can quantify these individual effects, and better understand the effect of the WPSH_SHO_3_ on the O_3_ pollution in Shanghai.

**Figure 10:**
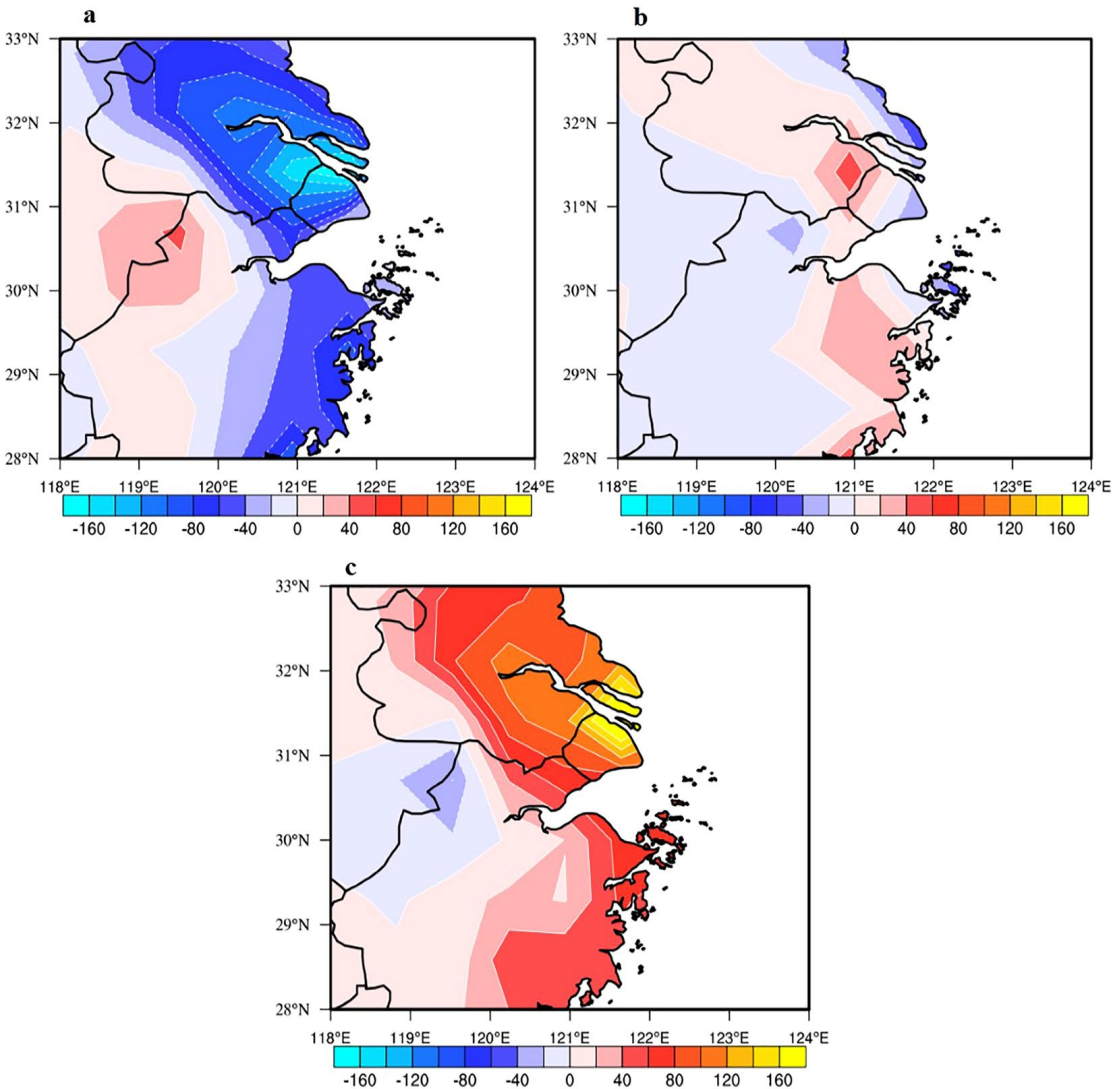
Anomalies of the contributions of individual processes to O_3_ formation (μg·m^−3^·day^−1^) in the Shanghai region in the strong and weak cases calculated by MOZART-4. The contributors include the (**a**) total diffusion (DIF), (**b**) advection (ADV) and (**c**) gas-phase chemistry (CHEM). The maps were generated using NCL software [The NCAR Command Language (Version 6.3.0) [Software]. (2015). Boulder, Colorado: UCAR/NCAR/CISL/TDD. 10.5065/D6WD3XH5].

Figure 10 shows the calculated anomalies of the contributions of individual processes to O_3_ formation in the Shanghai region under the different WPSH_SHO_3_ conditions. In the strong WPSH_SHO_3_ case, the vertical diffusion produced significant reduction for the O_3_ concentrations at the surface. This was due to the fact that with less cloud condition, the solar radiation was stronger, producing higher thermal turbulence and vertical diffusion. As a result, the surface O_3_ concentrations were vertical mixed in the upper planetary boundary layer, causing the decrease of O_3_ concentrations at surface layer in Shanghai (with a maximum reduction of 80–160 μg·m^−3^·day^−1^, shown in Fig. 10a). As we mentioned before, the wind direction in the strong case was favorable for the O_3_ concentrations, which resulted in about 20–40 μg·m^−3^·day^−1^ increase of surface O_3_ concentrations (Fig. 10b). The highest enhancement of O_3_ concentrations was due to the process of photochemistry. As shown in Fig. 10c, the maximum increase of O_3_ concentrations in Shanghai ranges about 100–180 μg·m^−3^·day^−1^, resulting from the higher O_3_ chemical productivity. In total, the surface O_3_ concentrations were higher in the strong WPSH_SHO_3_ case than the weak WPSH_SHO_3_ case in Shanghai.

## Conclusions

It is well known that WPSH is a strongest subtropical anticyclone high pressure system controlling monthly weather conditions in YRD during late-summer and may intensify in a warming climate background. To systemically study the impact of WPSH on inter-annual variability of ozone pollution in Shanghai under the climate change background, intensive surface measurements of ozone and meteorological data and the NCEP/NCAR re-analyzed meteorological data are used in the analysis. The highlights of this study are as follows:

1. To better understand the relationship between WPSH and O_3_ in Shanghai, we define geopotential height averaged over a key area (122.5°E-135°E, 27.5°N-35°N) at 500 hPa. In this region, the correlation coefficients between related to ozone concentration in Shanghai shows a maximum positive value (0.78). As a result, the WPSH’s in this region (defined as WPSH_SHO_3_) can better represent the effect of WPSH on the O_3_ concentrations in Shanghai, and are used in this study.
2. Long-term trend analysis shows that the WPSH_SHO_3_ index significantly increased from 1960 to 2017, with a rate of 5 gpm decade^−1^, resulting from the global climate change.
3. To quantify the effect of the increasing WPSH_SHO_3_ on the O3 concentrations in Shanghai, two cases (i.e., a weak WPSH_SHO_3_ case - occurred in Jul. 2017, and a strong WPSH_SHO_3_ – occurred in Aug. 2016) are selected and analyzed in this study. The results show that in the strong WPSH_SHO_3_ case, the lower relative humidity, higher temperature, less cloud, and higher solar radiation produced higher ozone photochemical production than the weak WPSH_SHO_3_ case. In addition the wind directions in the strong case were dominated by southwest wind. In the contrast, in the weak WPSH_SHO_3_ case, east and southeast winds were dominated, which transported relative clean air from ocean, resulting in lower O_3_ concentrations in the weak case.
4. Because there are several important factors in controlling the O_3_ concentrations, such as diffusion (DIF), (b) advection (ADV), and (c) gas-phase chemistry (CHEM), The MOZART model is applied to study the individual contributions to the surface O_3_ in Shanghai. The results show that in the strong WPSH_SHO_3_ case, the vertical diffusion produced significant reduction for the O_3_ concentrations at the surface. This was due to the fact that with less cloud condition, the solar radiation was stronger, producing higher thermal turbulence and vertical diffusion as well as the planetary boundary layer heights. As a result, the surface O_3_ concentrations were vertical mixed in the upper planetary boundary layer, causing the decrease of O_3_ concentrations at surface layer in Shanghai.

## Materials and Methods

### Measured surface chemical observations

Two measured surface O_3_ datasets are used in the study: (1) Hourly averaged O_3_ concentrations from 2013 to 2017 are measured by the Urban Air Quality Center in China, operated by the Chinese Environmental Production Ministration (CEPM), at 367 monitoring stations (http://106.37.208.233:20035). Based on the hourly average O_3_ concentrations, maximum average 8-hour ozone values (MDA8) and maximum average 1-hour ozone values (MDA1) are calculated in the Shanghai area. The monthly mean MDA8 O_3_ and MDA1 O_3_ concentrations from late-summer in 2013 to 2017 are further calculated. (2) The hourly photolysis rate of NO_2_ (J[NO_2_]) is measured and analyzed at the Pudong monitoring site of Shanghai Meteorological Service.

### Meteorological data

To make a comprehensive analysis of WPSH, four different meteorological data sets are used in this study: (1) The hourly surface meteorological parameters at Shanghai Baoshan Climate Observatory (including temperature, relative humidity, 10 m wind direction and wind speed) are measured and analyzed. The measurements in this site are used for the international meteorological data exchange sponsored by World Meteorological Organization (WMO). Thus these data can well depict the local meteorological characteristics in Shanghai. (2) Surface temperature at 160 Chinese monitoring sites are obtained from the National Climate Center of China. (3) The climate data of five WPSH indices (including area, intensity, ridge position, northern boundary position and western ridge point index) are obtained from the National Climate Center of China. (4) The large-scale weather conditions, such as general circulation are used the data from the National Center for Environmental Prediction (NCEP) and National Center for Atmospheric Research (NCAR) reanalysis data^41^. The data have a horizontal resolution of 2.5° × 2.5°, including the geo-potential height, specific humidity and winds data. In addition, the solar radiation data with a horizontal resolution of 1.875° × 1.904° is also used in this article.

### Global chemistry transport model (Mozart-4) description

A global chemistry transport model (MOZART-4; Model for Ozone and Related chemical Tracers, version-4) is used in this study. The detailed model description is shown by Emmons *et al*.^42^, and the detailed aerosol modules are shown by Tie *et al*.^43^. The MOZART-4 model is a global chemical transport model. The model is designed to study the global distributions of tropospheric trace gases and aerosol particles. In this study, the horizontal resolution of the model is 0.7° × 0.7°, with 42 vertical levels. Advection of tracers is performed using the flux-formed semi-Lagrangian advection scheme^44^. The deep convection scheme developed by Zhang and McFarlane^45^ is included in the model. The model transport is driven by the European Centre for Medium-Range Weather Forecasts (ECMWF) assimilated wind fields, with 0.5° × 0.5° horizontal resolution^46^. The meteorological data is interpolated to fit the model horizontal resolution by using a bilinear interpolation method. The MOZART model with such configurations has been successfully employed by Chang *et al*.^47^ to investigate the impact of El Niño event on regional air quality of China. The MOZART-4 model is applied to calculate the contributions of individual processes to O_3_ formation, including the total diffusion (DIF), advection (ADV) and gas-phase chemistry (CHEM).

## Data Availability

All data is available on-line and free of charge. NCEP Reanalysis data provided by the NOAA/OAR/ESRL PSD, Boulder, Colorado, USA, from their Web site at https://www.esrl.noaa.gov/psd/. The ECMWF ERA-Interim reanalysis data is available at the European Centre for Medium-Range Weather Forecasts. The WPSH indices (including area, intensity, ridge position, northern boundary position and western ridge point index) can be obtained from the National Climate Center of China. The observational data of surface O_3_ concentrations is available at http://106.37.208.233:20035. The photolysis rate of NO_2_ (J[NO_2_]) and surface meteorological parameters (including temperature, relative humidity, 10 m wind direction and wind speed) can be obtained from Shanghai Meteorological Service^48^.
